# Auditory Target and Novelty Processing in Patients with Unilateral Hippocampal Sclerosis: A Current-Source Density Study

**DOI:** 10.1038/s41598-017-01531-8

**Published:** 2017-05-09

**Authors:** Adrià Vilà-Balló, Clément François, David Cucurell, Júlia Miró, Mercè Falip, Montserrat Juncadella, Antoni Rodríguez-Fornells

**Affiliations:** 1 Cognition and Brain Plasticity Group [Bellvitge Biomedical Research Institute-IDIBELL], L’Hospitalet de Llobregat, Barcelona, 08097 Spain; 2 Dept. of Cognition, Development and Educational Science, Campus Bellvitge, University of Barcelona, L’Hospitalet de Llobregat, Barcelona, 08097 Spain; 3 Dept. of Psychology, Faculty of Education and Psychology, University of Girona, Girona, 17071 Spain; 4 Institut de Recerca Pediàtrica Hospital Sant Joan de Déu, Barcelona, Spain; 5 Epilepsy Unit, Neurological Service, Hospital Universitari de Bellvitge, L’Hospitalet de Llobregat, Barcelona, 08907 Spain; 6 Catalan Institution for Research and Advanced Studies, ICREA, Barcelona, Spain

## Abstract

The capacity to respond to novel events is crucial for adapting to the constantly changing environment. Here, we recorded 29-channel Event Related Brain Potentials (ERPs) during an active auditory novelty oddball paradigm and used for the first time Current Source Density-transformed Event Related Brain Potentials and associated time-frequency spectra to study target and novelty processing in a group of epileptic patients with unilateral damage of the hippocampus (N = 18) and in healthy matched control participants (N = 18). Importantly, we used Voxel-Based Morphometry to ensure that our group of patients had a focal unilateral damage restricted to the hippocampus and especially its medial part. We found a clear deficit for target processing at the behavioral level. In addition, compared to controls, our group of patients presented (i) a reduction of theta event-related synchronization (ERS) for targets and (ii) a reduction and delayed P3a source accompanied by reduced theta and low-beta ERS and alpha event-related synchronization (ERD) for novel stimuli. These results suggest that the integrity of the hippocampus might be crucial for the functioning of the complex cortico-subcortical network involved in the detection of novel and target stimuli.

## Introduction

The capacity to respond to novelty is crucial for adapting to the constantly changing environment. During the last decades, non-invasive event-related brain potentials (ERPs) collected during classic oddball paradigms or active oddball tasks have been used to investigate the electrophysiological correlates of novelty processing in humans. In oddball paradigms, novel stimuli elicit a fronto-central P3a ERP component peaking around 250–350 ms post-stimulus onset^1, 2^. This ERP component has been suggested to be an index of novelty processing and attentional switching^2^. In active oddball tasks, two different components are generally observed. On the one hand, the “novelty” P300 is elicited by non-target distractors^3–6^ and may reflect the reorientation of attention^7–9^. On the other hand, target stimuli elicit a centro-parietal P3b ERP component peaking about 300–600 ms post-stimulus onset which is rather related to subsequent contextual memory comparisons required to provide a behavioral response^10^. Importantly, both the P3a and the “novelty” P300 components may reflect the involvement of frontal attentional processes related to the orienting response^2, 11^.

A large body of research in humans and animals has demonstrated that the hippocampus, known to be involved in associative and spatial memory processes, is also essential for target and novelty processing^12–25^. Following these lines, the link between the integrity of the hippocampus and auditory novelty processing in humans was first reported in an electroencephalography (EEG) study showing that damage to hippocampal and surrounding tissues due to stroke at the posterior cerebral artery selectively attenuated the amplitude of the P3a component to novel items but not the P3b amplitude to targets in an active auditory oddball task^26^. Moreover, the integrity of the hippocampus does not predict the amplitude of the P3b component to targets in temporal-lobe epileptic patients with unilateral sclerotic hippocampus (TLE-UHS)^27^. Confirming this, a single-case study in a patient with bilateral hippocampal damage revealed that the hippocampus seems not to be indispensable for the generation of the P3b component but that the latency is clearly affected by hippocampal lesions^28^. However, even if it is still a debate as to whether the scalp EEG contains any hippocampal signal at all, it seems possible to find zero-phase lag correlation between hippocampal activity and Magneto-Encephalographic (MEG) scalp activity^29^. There is also recent evidence showing that hippocampal oscillatory activity in the theta band can be observed in MEG^30^. In addition, the simultaneous recordings of scalp and intracranial EEG activity during auditory and visual oddball tasks converge in showing that the hippocampus is involved in the generation of surface P3 component^31, 32^.

Current Source Density transformations of the EEG activity (CSD-transformed EEG) have been used to study novelty processing in healthy participants. This approach, also known as Laplacian transformed EEG (LT-EEG), is commonly used as a reference-free method to sharpen ERP topographies in a physiologically meaningful manner^33, 34^. Recent evidence shows that CSD transformation allows establishing a more reliable link between the electrophysiological activity and the underlying cognitive processes^35–38^ and importantly, that the auditory P3a and P3b components could be clearly disentangled using LT-EEG^39^. Recently, Tenke and colleagues^40^ have used CSD transformations of the EEG combined with a principal components Analysis (PCA) to compare a group of depressed patients to healthy controls in an auditory novelty oddball task. Compared to controls, depressed patients exhibited an attenuated P3a source for novel stimuli and importantly, this component was preceded by the so-called Novelty Vertex Source (NVS) which was also attenuated in patients. Interestingly, the topographical distribution of the NVS suggested the contribution of deep generators. However, very few studies have focused on auditory novelty processing in epileptic patients^41^ and importantly, LT-EEG decomposition has not been used until now. The analysis of event-related oscillations (EROs) and Time-Frequency (TF) transforms of Current Source Density activity have also been employed to assess novelty processing in healthy participants showing (i) a consistent increase of theta and low-beta power for novel or distractive stimuli, (ii) an increase of theta power for target stimuli^42^. Importantly, a recent study compared individuals at clinical high risk for psychosis to matched control participants in a three-stimulus auditory novelty oddball task^43^. The authors performed a PCA on the TF transforms of the CSD data to accurately delineate the TF components of novelty and target processing. While targets elicited a clear alpha ERD over posterior regions coinciding with the P3b source time interval, novel stimuli elicited two different theta ERS, one during the N1 sink interval and the other during the P3a source interval. Interestingly, compared to controls, patients presented an attenuation of alpha ERD to targets but a similar pattern of CSD-transformed ERPs to novel stimuli. These results are in line with previous findings showing an increase of alpha ERD during an active auditory oddball task as compared to a passive condition^44^.

The main goal of the present study was to explore the electrophysiological correlates of target and novelty processing in patients with unilateral hippocampal sclerosis. With this aim and following previous reports^26, 41^, we used for the first time CSD-transformed ERPs and associated TF spectra to compare a group of epileptic patients with unilateral damage restricted to the hippocampus to a group of healthy matched control participants. Importantly, the clinical sample was homogeneous with respect to the volume of tissue damage, which allows a better exploration for the functional role of this structure. Moreover, to further study the role of the lesion side as well as the possible effect of GABAergic medication, we divided our group of patients into different sub-groups and performed the corresponding comparisons. Considering previous EEG studies in humans^26, 27^, we expected to find a reduction in the LT-EEG ERPs associated to the novelty P3a in patients compared to controls. Based on previous animal studies using single unit recordings and showing that novel stimuli induce bursts of theta oscillatory activity within the hippocampal-Ventral Tegmental Area (VTA) dopaminergic loop^19, 45, 46^, we expected to observe a modulation of the EROs to novel stimuli associated to a damaged hippocampus. Specifically, compared to the control group we expected to observe a reduction of theta and/or low-beta ERS in our group of patients. Because there is mixed available evidence in the literature for the involvement of the hippocampus in target processing, we were not expecting to observe clear differences between groups in any of the brain measures collected related to target processing. Within the group of patients, we were not expecting a clear effect of lesion side but a negative effect of GABAergic medication on these measures.

## Results

### Behavioral results

Reaction times (RTs) for target stimuli were obtained. For each participant, RTs that were +/− 3 SD outside the individual mean were excluded from the analyses. The number of trials removed were not different between the two groups [Control: 2.60 ± 0.77%, TLE-UHS: 2.889 ± 1.11%; *t*(30) = −0.855, *p* = 0.399]. Four patients were excluded from the RTs analyses because they had less than 20 correct responses. This exclusion criterion was based on previous literature^47^, showing that a minimum of 20 correct trials is needed to obtain a reliable ERP component. Results of this analysis showed that the control group was faster for target tones (494.2 ± 66.5 ms) than the group of remaining patients (551.6 ± 85.7 ms; *t*(30) = −2.133, *p* = 0.041). Similarly, the percentage of non-responded trials, which was obtained by dividing the number of non-responded trials by the number of responded trials, was significantly lower in controls (12.0% ± 13.8) than in patients (35.2% ± 38.2; *t*(34) = −2.421, *p* = 0.021). Importantly, the percentage of responses in non-target trials, which was obtained by dividing the number of non-target trials with responses by the total number of non-target trials, was not significantly different between controls (9.3% ± 12.2) and patients (12.6% ± 6.8; *t*(34) = −1.007, *p* = 0.321). Similar results were obtained when discarding the patients with low target detection performance (see Supplementary information). Finally, no differences were observed between the left and right TLE-UHS patients in any of the behavioral measures but we found that patients with GABAergic medication were slower than those without this medication (see Supplementary information).

### Voxel-Based Morphometry results

**Figure 1:**
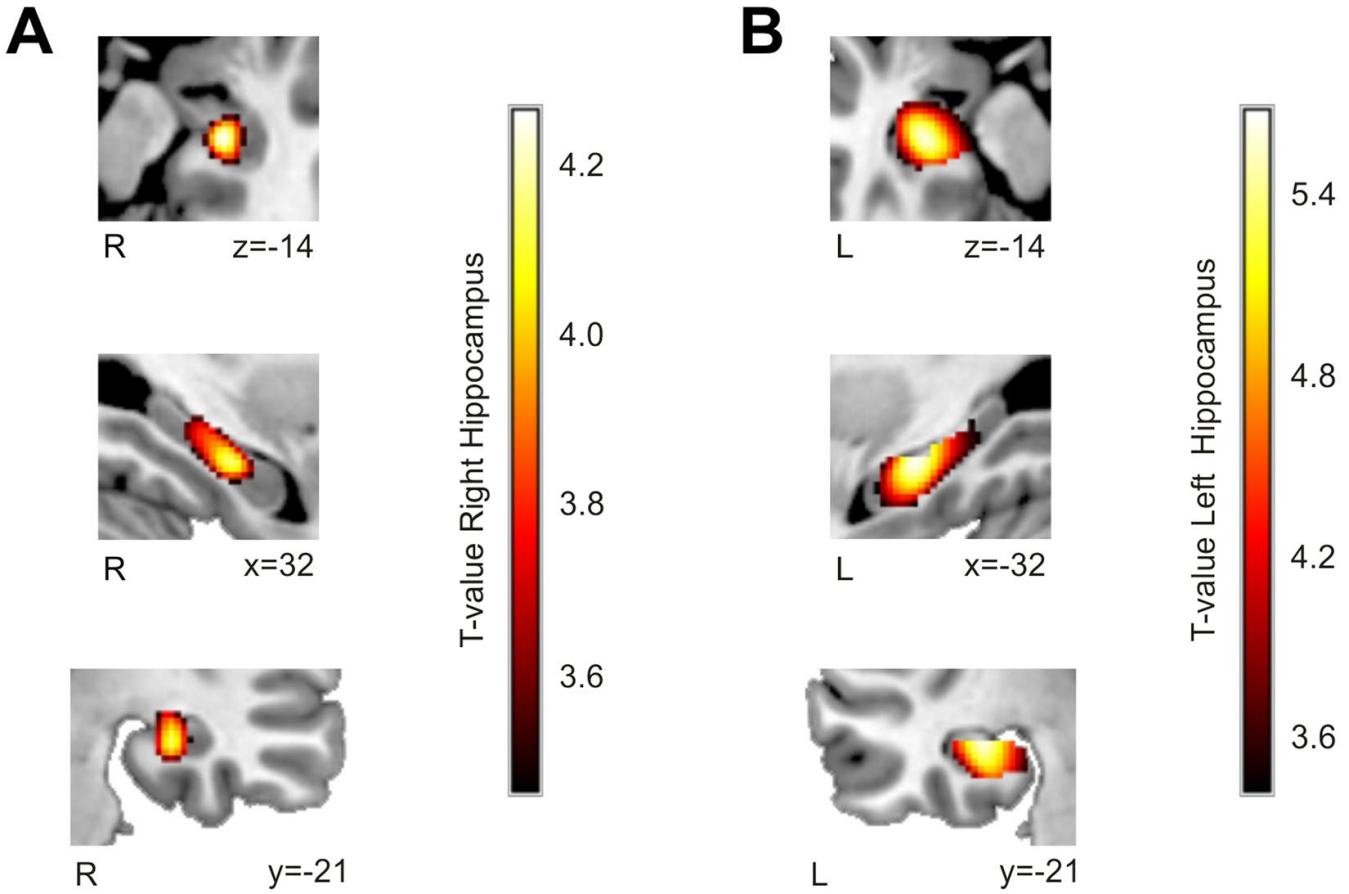
Voxel-Based Morphometry comparison between groups: (**A**) control > right TLE + UHS patients and (**B**) control > left TLE + UHS. Both groups of patients showed a decreased hippocampal grey matter compared to healthy participants. Results are shown in red-yellow at an auxiliary *p* < 0.005 uncorrected threshold at the voxel level (main peaks in both clusters survived a Family-wise error Correction for Small Volume *p* < 0.05 threshold). Neurological convention is used with Montreal Neurological Institute (MNI) coordinates at the bottom right of each slice. R, Right Hemisphere; L, Left hemisphere.

Figure 1 shows the results of the Voxel-Based Morphometry (VBM) analysis comparing the volume of Grey Matter (GM) in the hippocampus of the two groups of patients and the control group. The right TLE-UHS patients showed, as expected, decreased GM volume in the right hippocampus [one cluster with 580 voxels; x = 30, y = −18, z = −15; *t*(25) = 4.27, *p* < 0.011 Family-Wise Error corrected using Small Volume Correction; see Fig. 1A] when compared with the control group. Similarly, left TLE-UHS patients showed decreased GM volume in the left hippocampus when compared to controls [one cluster with 1673 voxels; x = −30, y = −19, z = −12; *t*(25) = 5.68, *p* < 0.001 Family-Wise Error corrected using Small Volume Correction; see Fig. 1B]. These results further confirm that the TLE-UHS group presented less hippocampal GM volume than controls. The decrease of GM mainly affected the medial part of the hippocampus. Importantly, based on visual inspection, the lesion seemed to be restricted to this structure with very few damage to surrounding issues.

### Electrophysiological results

#### Time analysis of CSD waveforms

In order to better isolate the neural correlates of target and novelty processing, the difference waveforms were obtained by subtracting the CSD components elicited by the standards from those elicited by target and novel stimuli. The difference waveforms (target-standard time-window: 470–570 ms; novel-standard time-window: 270–370 ms) were used for statistical analyses. The size of the time-windows were determined accordingly to Marco-Pallarés *et al*.^42^. Additionally, for each participant, we computed (i) the peak-to-peak amplitude (for target stimuli: between N2 sink and the P3b source; for novel stimuli: between N2 sink and the P3a source) and, (ii) the peak latency in the electrode showing maximum amplitude (Pz for P3b source and Fz for the P3a source).

**Figure 2:**
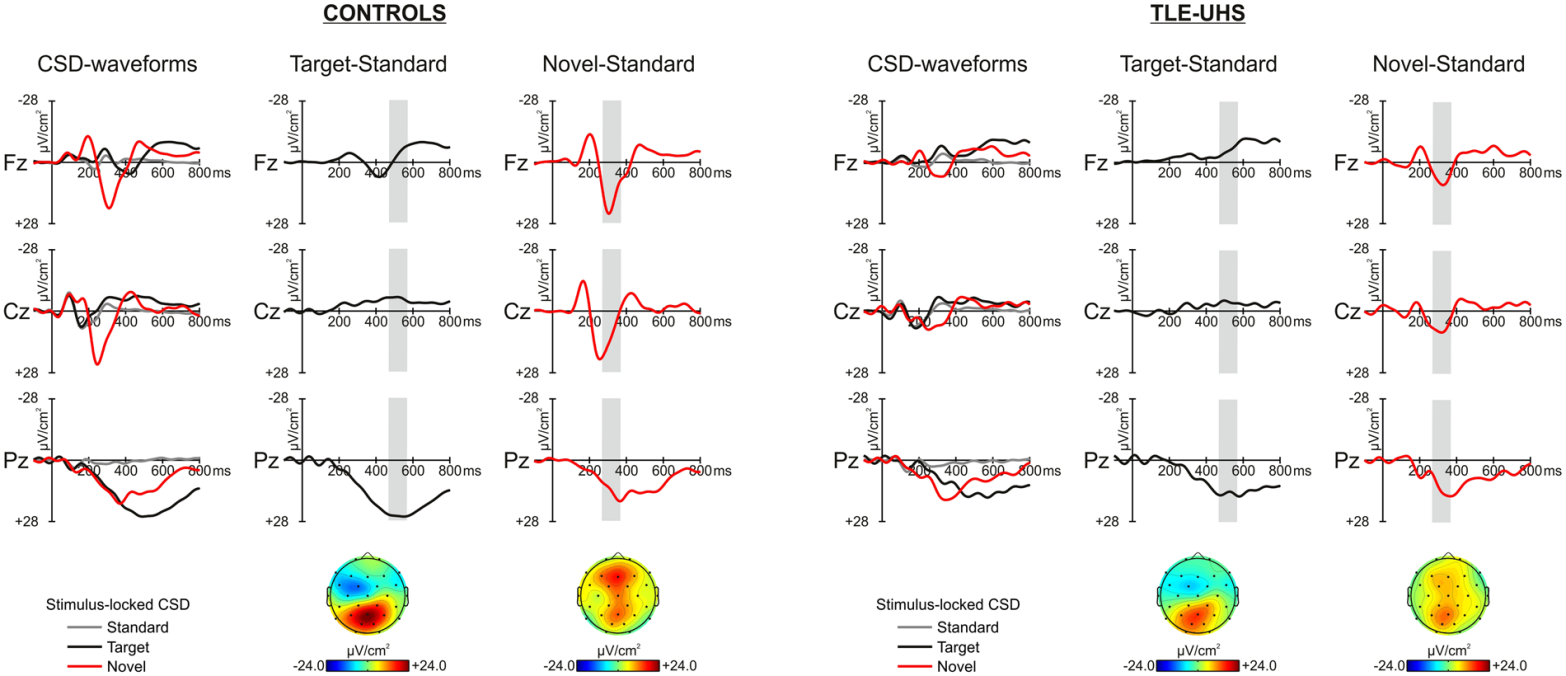
Grand mean CSD waveforms for standard (grey line), target (black line) and novel (red line), at midline electrodes (Fz, Cz, and Pz), from –100 to 800 ms, for both the control and the TLE-UHS group. Grey areas indicate the time-windows considered for the analyses. Difference waveforms associated to the target minus standard (black line) and novel minus standard (red line) are showed. Bottom part: scalp distribution of the P3b source (Target minus standard, −24/24 μV/cm^2^), and of the P3a source (Novel minus standard, −24/24 μV/cm^2^).

As shown in Fig. 2, both groups exhibit similar CSD-transformed ERP components associated to the classical P3b source for targets. As found in previous studies^39, 43^, novel stimuli elicited the Novelty Vertex Source (NVS) followed by the fronto-central P3a source. These CSD components appeared attenuated in patients as compared to controls. The grand mean difference waveform of CSD activity between targets and standards shows a clear deflection at around 520 ms post-onset maximum at Pz electrode in both groups. Despite the fact that this component may appear larger in controls than in patients, results of the ANOVA failed to show significant differences between the two groups (main effect of group: *F*(1,34) = 2.830, *p* = 0.15; group x electrode interaction: *F*(2,68) = 1.713, *p* = 0.19, ε = 0.933). Besides this, no differences between the two groups were found in the peak-to-peak analyses either for the mean amplitude (Control: 34.00 ± 12.49 μV/cm^2^, TLE-UHS: 30.66 ± 11.72 μV/cm^2^; *t*(34) = 0.827, *p* = 0.414) or for the latency (Control: 515.17 ± 70.45 ms, TLE-UHS: 526.44 ± 62.21 ms; *t*(34) = −0.509, *p* = 0.614).

In sum, CSD-transformed ERP components to target stimuli were not different between the groups. Importantly, similar results were obtained when discarding the four patients with low target detection performance (see Supplementary information and Fig. S1). Besides, no differences were observed in the group of patients when taking into account the side of the lesion or GABAergic medication (see Supplementary information and Figs S2 and S3).

The grand mean difference waveform of CSD activity between novel and standard stimuli shows a clear fronto-central deflection, peaking at around 320 ms post-stimulus onset. Results of the ANOVA revealed that controls had a larger amplitude than patients across all electrodes (significant main effect of group: *F*(1,34) = 4.171, *p* = 0.049; group x electrode interaction: *F*(2,68) = 1.419, *p* = 0.249, ε = 0.968). Similar results were found in the peak-to-peak analyses for the peak amplitude (Control: 49.64 ± 25.89 μV/cm^2^, TLE-UHS: 35.26 ± 13.19 μV/cm^2^; *t*(34) = 2.101, *p* = 0.046) as well as for the peak latency with patients showing a reduced and delayed response as compared to controls (Control: 310.89 ± 43.19 ms, TLE-UHS: 339.78 ± 41.69 ms; *t*(34) = −2.042, *p* = 0.049). No group differences were also observed after discarding the patients with low target detection performance (see Supplementary information and Fig. S1). Importantly, no differences were observed in the group of patients when taking into account the side of the lesion. However the patients receiving GABAergic medication exhibited a delayed response as compared to patients without medication (see Supplementary information and Figs S3 and S4).

### Time-Frequency analysis of CSD waveforms

**Figure 3:**
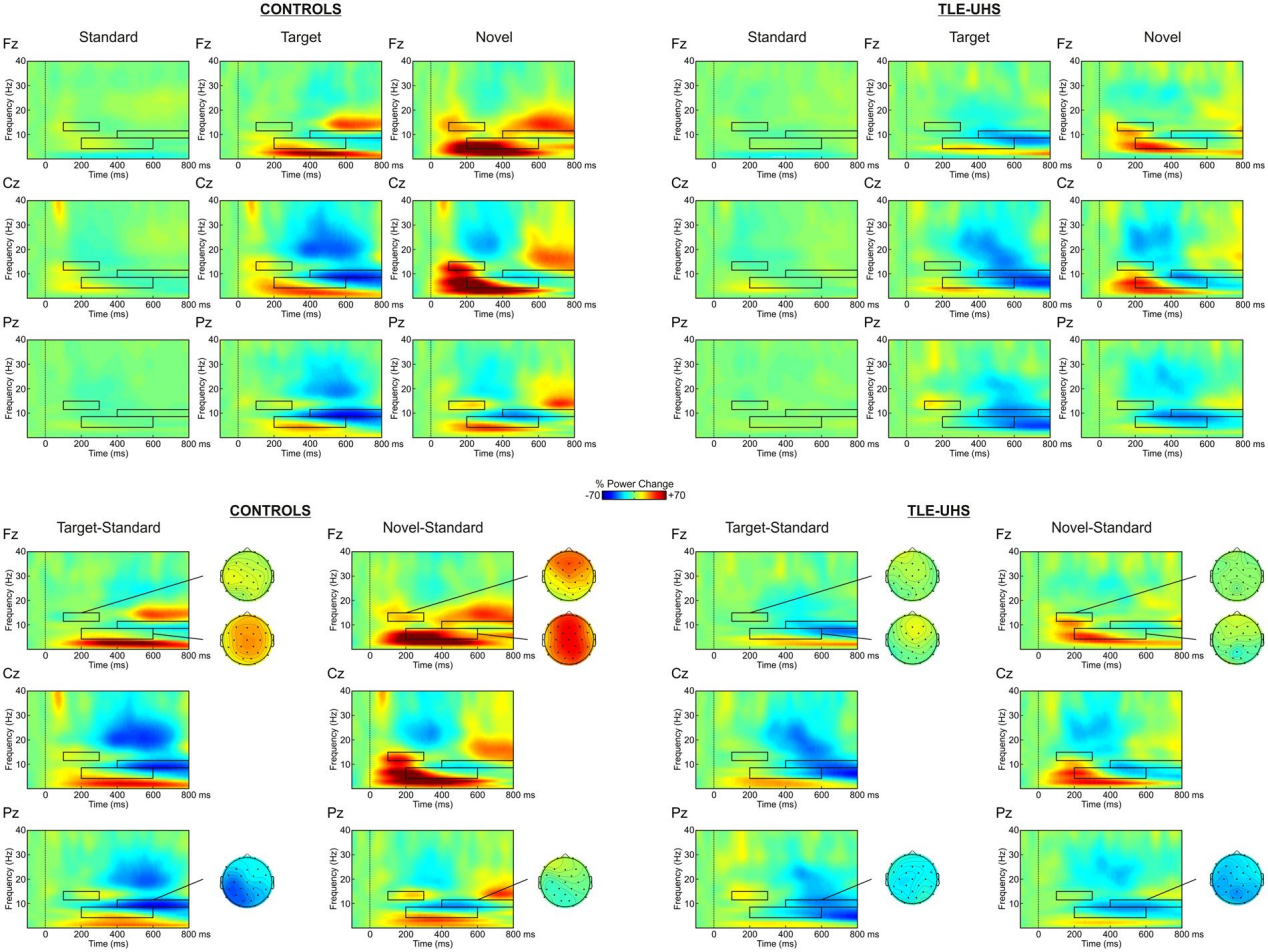
Grand mean CSD event-related spectral perturbation representing changes in power with respect to baseline of standard, target, and novel stimuli at midline electrodes, for the control (left) and the TLE-UHS group (right). The increase/decrease of power is represented from −100 to 800 ms. The black squares indicate the time-windows in the different frequency bands considered for the analyses. Note that standard stimuli elicited significant low-beta ERS and alpha ERD when compared to the baseline. These differences are clearly visible with a reduced scale. Differences in power between target minus standard, and between novel minus standard are depicted at the bottom of the figure. The power distributions of the theta (4–8 hz), alpha (8–12 hz), and low-beta (12–15 hz) activities for target minus standard and for novel minus standard are depicted.

Figure 3 shows the Event-related Spectral Perturbations plots for the different conditions. Targets and novel stimuli elicited clear theta ERS when compared to baseline (200–500 ms, 4–8 Hz, *F*(1,34) = 9.429, *p* = 0.004, and *F*(1,34) = 20.409, *p* < 0.001). This was not the case for standard stimuli (*F*(1,34) = 0.358, *p* = 0.554). Compared to baseline, standard and novel stimuli elicited significant low-beta ERS (100–300 ms, 12–15 Hz, *F*(1,34) = 12.005, *p* = 0.001, and *F*(1,34) = 6.644, *p* = 0.014) but targets did not (*F*(1,34) = 0.038, *p* = 0.846). Compared to baseline, significant alpha ERDs (400–800 ms, 8–12 Hz) were observed for target (*F*(1,34) = 58.5118, *p* < 0.001), novel (*F*(1,34) = 21.720, *p* < 0.001) as well as for standard stimuli (*F*(1,34) = 16.473, *p* < 0.001). The corresponding time-windows and frequency bands were used for statistical analyses of the difference waveforms. Indeed, in order to directly compare the two groups, we computed the differences of power in the theta, low-beta, and alpha bands (i) between target stimuli and standards and (ii) between novel stimuli and standards (see Fig. 3). Interestingly, the target and novelty effects appeared to be attenuated in patients.

For target stimuli, results of the ANOVA revealed that patients exhibited lesser theta ERS than controls over all electrodes (significant main effect of group: *F*(1,34) = 23.235, *p* < 0.001; group x electrode interaction; *F*(2,68) = 3.472, *p* = 0.063, ε = 0.588). No significant differences between the two groups were found for low-beta ERS (main effect of group: *F*(1,34) = 1.991, *p* = 0.167; group x electrode interaction: *F*(2,68) = 0.324, *p* = 0.634, ε = 0.659) and alpha ERD (main effect of group: *F*(1,34) = 0.005, *p* = 0.943; group x electrode interaction: *F*(2,68) = 0.535, *p* = 0.507, ε = 0.623). Importantly, similar results were obtained when discarding the patients with low target detection performance (see Supplementary information and Fig. S4). No differences were observed in the group of patients when taking into account GABAergic medication. However, we found that patients with a left lesion exhibited greater alpha ERD than patients with a right lesion (see Supplementary information and Figs S5 and S6).

For novel stimuli, patients presented an attenuated theta ERS compared to controls over all electrodes (significant main effect of group: *F*(1,34) = 24.352, *p* < 0.001; group x electrode interaction: *F*(2,68) = 1.133, *p* = 0.311, ε = 0.678). Similarly, patients presented an attenuated low-beta ERS compared to controls (significant main effect of group: *F*(1,34) = 9.616, *p* = 0.004 and group x electrode interaction: *F*(2,68) = 3.490, *p* = 0.049, ε = 0.771). This difference was largest at Fz electrode (Fz: *t*(34) = 3.305, *p* = 0.003; Cz: *t*(34) = 2.769, *p* = 0.010; Pz: *t*(34) = 2.560, *p* = 0.017). Patients also presented lesser alpha ERD than controls (significant main effect of group: *F*(1,34) = 5.632, *p* = 0.023; group x electrode interaction: *F*(2,68) = 4.746, *p* = 0.026, ε = 0.654). This difference was significant over fronto-central electrodes (Fz: *t*(34) = 3.272, *p* = 0.003; Cz: *t*(34) = 2.365, *p* = 0.024; Pz: *t*(34) = 1.187, *p* = 0.243). As found for target stimuli, similar results were obtained when discarding the patients with low target detection performance (see Supplementary information and Fig. S4). No differences were observed in the group of patients when taking into account the side of the lesion or medication (see Supplementary information and Figs S5 and S6).

## Discussion

In the present study, we used CSD-transformed ERPs and associated time-frequency spectra to compare a group of temporal-lobe epileptic patients with unilateral sclerotic hippocampus to a group of healthy participants during an active novelty auditory oddball task. Importantly and compared to previous studies^26, 41^, we used VBM to ensure that our group of patients had a focal unilateral damage restricted to the hippocampus and especially its medial part. Specifically, we found a higher number of misses and slower RTs in the TLE-UHS group than in the control group suggesting a deficit for target processing at the behavioral level. In addition, compared to controls, our group of patients presented (i) a reduction of theta ERS for targets and (ii) an attenuated and delayed NVS/P3a sources, theta and low-beta ERS and alpha ERD for novel stimuli. These last results suggest both target and novelty-processing deficits in patients with TLE-UHS.

Concerning target stimuli, while previous ERP studies on TLE patients revealed no effect on the P3b, suggesting that the hippocampus may not contribute to the scalp activity related to target processing^26, 48, 49^, intracranial recordings and functional Magnetic Resonance Imaging (fMRI) data showed the opposite with a clear involvement of the hippocampus in target processing^12–16, 25, 31, 32^. Recent studies using combined intracranial EEG recordings and fMRI in epileptic patients during an auditory oddball tasks have revealed that the P3 is not an homogeneous component originating from one single brain region but that multiple cortical and mesiotemporal structures are involved in the generation of the surface potential^50, 51^. In the present study, TLE-UHS patients presented behavioral evidence for a deficit in target processing with more misses and slower RTs than controls. Importantly, this behavioral deficit was accompanied by an attenuated Theta ERD in the TLE-UHS group compared to controls. A possible explanation for the different results obtained may be due to differences in perceptual saliency of the stimuli used in the previous studies. It may also be the case that EROs are more sensitive than classic ERP analyses to capture the smaller differences between the different type of deviant stimuli. Indeed, despite the fact that it still remains unclear whether surface EEG recordings can reflect hippocampal activity, recent evidence suggests that memory integration is predicted by the increase of theta coherence between the hippocampus and middle prefrontal cortex^52^. However, we cannot draw a clear conclusion on the direct contribution of hippocampal damage to the present EEG results. Indeed, we did not observe clear associations between the hippocampal grey matter damage (measured using VBM) and the main ERPs/EROs effects. The link between hippocampal damage and the attenuation of the EEG features reported here might need further research, most probably including larger samples of both unilateral and bilateral sclerotic hippocampus patients^53^ or combining simultaneous recordings of intra-cortical sEEG with surface EEG/MEG in epileptic patients^54, 55^.

Regarding novel stimuli, the use of CSD-transformed ERPs allowed us observing the NVS/P3a sources in patients with TLE-UHS which confirms the results of previous studies with other pathological populations^40, 43^. As reported in these studies, the topographical distribution of the NVS/P3a source suggests the contribution of deep generators. Besides this, previous studies using fMRI and positron emission tomography studies in healthy participants with different tasks and modalities have consistently revealed hippocampal activation during novelty processing^22, 56–61^. Unilateral hippocampal damage has been previously shown to induce a decrease of the P3a amplitude for novel stimuli presented in the auditory and tactile modalities^26^. However, most of the patients from the previous study had lesions induced by an infarction affecting a large portion of the temporal lobe, thus rendering difficult to determine the role of the hippocampus in novelty processing. By contrary, our group of patients presented more focal lesions of the hippocampus, with very few damaged surrounding tissue (see results of the VBM analysis). This was accompanied by a reduction of all the electrophysiological measures associated to novelty processing in the TLE-UHS group as compared to the control group with no difference between patients with left or right lesions (see Supplementary information). Importantly, it has been revealed using intracranial recordings that patients with TLE due to hippocampal sclerosis exhibit a smaller N400 than controls without hippocampal sclerosis suggesting a deficit of novelty processing for linguistic material^20^. Our results extend those previous data by showing that TLE-UHS patients can also exhibit altered brain signatures of novelty-processing deficit for non-linguistic stimuli.

Interestingly, it has been suggested that the hippocampus may compare the incoming information and contribute to the detection of mismatch between novel and familiar events^62–67^. There is also evidence showing that a multimodal mechanism used to detect novel stimuli might be used to detect deviant non-novel stimuli as well^25^. Therefore, a similar mechanism might be used to detect both novel and non-novel deviant stimuli. The continuous comparison/detection mechanisms of specific features or combination of features might also be the basic neurophysiological processes underlying working memory maintenance^68, 69^. Indeed, a previous study using fMRI in healthy participants have demonstrated that novelty-related activations of the hippocampus during working memory maintenance of faces predicted long-term memory performance^68^. Moreover, an intracranial EEG study in epileptic patients suggests that the dynamic of alpha and delta oscillations recorded from the hippocampus predicted performance of working memory maintenance of faces^70^.

The current findings may present some limitations related to the intake of GABAergic medication in our group of patients. Indeed, previous pharmacological studies have revealed mixed effects of GABAergic agonist and antagonists on pre-attentive and attentive ERP indices of auditory novelty processing^71–75^. While GABAergic agonists such as Benzodiazepines generally reduce the amplitude and increase the latency of ERP components related to novelty detection^74, 75^ (but see Kasai *et al*.^73^ for different results), GABAergic antagonists such as Flumazenil seem to increase attentional processes^72^. Here, we found a complex pattern of results with patients under GABAergic medication showing slower RTs and delayed brain response to targets as compared to non-medicated patients. However, we found no differences in the amplitude of the CSD components related to target processing. We did not observe significant differences on any of the target and novelty-related EROs either. Therefore, it is unlikely that the differences observed between patients and controls may be explained by the presence of GABAergic medication in our group of patients. Another limitation of the present study might be about the non-specificity of the deficits that we have observed here. Indeed, the entire group of patients showed worse performance on several neuropsychological tests of verbal short-term memory. Nonetheless, the comparison including the patients with good behavioral responses shows that most of the differences do not hold anymore thus suggesting that a general attentional or cognitive deficit may not explain the differences.

To conclude, the current study provides complementary behavioral and electrophysiological evidence for novelty and target deficits in patients with TLE-UHS. Importantly, our group of patients presented a focal lesion of the medial hippocampus (partially affecting the left anterior and posterior hippocampus) differing from previous studies in which lesions affected the surrounding temporal regions. Future research should be performed to determine whether scalp EEG recordings like the ones gathered here directly reflect hippocampal activity or rather the modulation of distant cortical sources involved in novelty and target processing.

## Methods

### Participants

A total of eighteen pharmaco-resistant temporal lobe epilepsy patients with unilateral sclerotic hippocampus (TLE-UHS) (nine men, mean age: 39.72 ± 10.64; mean years of education: 12.61 ± 3.05) participated in the study. Half of the patients had left and the other half had right temporal lobe epilepsy. The patients were recruited at the University Hospital of Bellvitge after presurgical evaluation. The diagnosis was based on clinical EEG and magnetic resonance imaging (MRI) data^76–78^. All patients underwent a neurological and standardized neuropsychological examination, prolonged interictal and ictal video-EEG monitoring, and brain MRI assessed by both a neurologist and a neuro-radiologist. All the patients presented no sign of seizure during the study or 24 hr before. All of them were taking anti-seizure medications at the time of testing. Additionally, a group of eighteen healthy participants were recruited for the purpose of the study (nine men, mean age: 39.17 ± 11.32; mean years of education: 12.33 ± 3.22). The two groups were matched for gender (*U* = 162, *Z* = 0.000, *p* = 1.000), age (*t*(34) = −0.152, *p* = 0.880), years of education (*t*(34) = −0.266, *p* = 0.792), and handedness (*t*(34) = 0.586, *p* = 0.562). For all participants, the neuropsychological examination was carried out before the MRI session. The demographic and neuropsychological data are described in the Supplementary Information. Written informed consent was obtained before the experiment from all of the participants. The study was carried out in accordance with the guidelines of the Declaration of Helsinki (BMJ 1991; 302: 1194), and approved by the Ethical Committee of University Hospital of Bellvitge, Spain.

### Paradigm

We used a variant of the auditory active oddball paradigm^42^ in which an infrequent target tone (1620 Hz, 60 ms duration, 5-ms rise/fall times) occurred with a probability of *P* = 0.2 in a stream of standard tones (1500 Hz, 60 ms duration, 5-ms rise/fall times) which occurred with a probability of *P* = 0.6. In addition of standard and infrequent target tones, novel sounds (short excerpts of environmental sounds such as the barking of a dog or the honking of a car) were also presented with a probability of *P* = 0.2 (average duration: 60.95 ± 7.61 ms). The stimuli were presented binaurally through headphones at 75 dB SPL in pseudo-random order with a stimulus onset asynchrony set to 1200 ms ( ±100 ms). A total of 500 trials were presented over 5 blocks of 100 trials with 60 standards tones. The participants were instructed to respond as quickly and accurately as possible to the target tones with the right index finger, and to ignore standard and novel tones.

### Electrophysiological Recording

The EEG activity was recorded continuously (digitized with a sampling rate of 250 Hz, 0.01 Hz high-pass filter and 50 Hz notch filter) using 29 tin electrodes, mounted in an elastic cap and located at standard positions (Fp1/2, F3/4, C3/4, P3/4, O1/2, F7/8, T3/4, T5/6, Fz, Cz, Pz, Fc1/2, Fc5/6, Cp1/2, Cp5/6, Po1/2). The EEG was referenced on-line to the right ocular canthus. Biosignals were re-referenced offline to the mean of the activity at the two mastoid processes. Electrode impedances were kept below 5 kΩ. Vertical eye movements were monitored by an electrode placed at the infraorbital ridge of the right eye.

### Data analysis

ERPs were obtained separately for standard, target and novel tones (from −100 until 1000 ms post-stimulus onset) and were baseline-corrected from −100 ms until 0 ms post-stimulus onset, as done in previous studies^42, 43^. Epochs exceeding ±100 µV in electro-oculogram (EOG) or EEG were automatically detected and removed from further analysis after confirmation by visual inspection. Due to the low number of correct responses for target tones in the TLE-UHS group, responded and non-responded target trials were included in the average. Supplementary analyses were also performed with a reduced group of patients (N = 14) including only the correct trials (see Supplemental information).

The EEG signal was transformed into reference-free CSD waveforms using the spherical spline surface Laplacian algorithm with fourth-degree Legendre polynomials and a smoothing coefficient (λ value) of 10^−5^ ^79^. The CSD waveforms were extracted from each original ERP waveform using a CSD toolbox for MATLAB^34^. These estimates represent the radial current flow entering and leaving the scalp and are proportional to the direction, location, and intensity of current generators that underlie an ERP map^35, 80, 81^.

Similarly, the associated frequency spectra elicited by standard, target and novel tones were also obtained (from −2000 ms until 2000 ms post-stimulus onset). Epochs exceeding ± 100 µV in EOG or EEG were automatically detected and removed from further analysis after confirmation by visual inspection. As done for the CSD components, the baseline was located in the 100 ms preceding the stimulus^42^ and the target waveforms for the TLE-UHS group included both responded and non-responded target trials (but see Supplementary information for additional analyses). Single trial CSD data was convoluted using a 7-cycles complex Morlet wavelet^82, 83^. Changes in time varying energy (square of the convolution between wavelet and signal) in the studied frequencies (from 1 Hz to 40 Hz; linear increase) with respect to baseline were computed for each trial and averaged for each participant before performing the grand-average. The analyses were carried out by transforming the time-frequency epochs into reference-free CSD event-related spectral perturbation using exactly the same procedure described above for the CSD waveforms^79–81^. The mean percentage of rejected epochs was 10.54% ± 7.91 for the control and 27.43% ± 17.01 for the TLE-UHS group. Overall, the TLE-UHS group had significantly more artifacts than the control group (*F*(1,34) = 14.6, *p* < 0.001) but this effect was equally distributed across conditions with no significant interaction between group, and the percentage of rejected epochs in each condition (*F*(2,68) = 0.5, *p* = 0.58).

### Statistical analysis

We used two-sample *t*-tests to compare the two groups (Control, TLE-UHS) for each of the behavioral measures.

For the time analysis of CSD waveforms, the individual difference waveforms between target and standard stimuli, and between novel and standard stimuli were obtained. As done in Marco-Pallarés *et al*.^42^, the mean amplitudes of the target-related P3b source and novelty-related P3a source were set at ± 50 ms centered on the peak activity of each component. Similarly, theta ERS (200–500 ms, 4–8 Hz), alpha ERD (400–800 ms, 8–12 Hz) and low-beta ERS (100–300 ms, 12–15 Hz) time-windows and frequency ranges were defined based on previous literature^42, 43^. Specifically, we analyzed the CSD components and TF measures separately for target and novel stimuli. In all the cases, the mean amplitude of the difference waveforms in the selected time-windows were submitted to a repeated-measures ANOVAs with Electrode location (Fz, Cz, Pz) as within-subject factor and Group (Control, TLE-UHS) as between-subject factors. In addition, we used two-sample *t-*tests to compare the peak-to-peak amplitudes and peak latencies between the groups (see Results section for details). All *P* values reported below were corrected using the Greenhouse-Geisser correction for nonsphericity when appropriate^84^.

### MRI data acquisition

Whole-brain structural MRI scans including T1-weighted and FLAIR images were acquired from both controls and TLE-UHS patients. The high-resolution T1-weighted images (slice thickness = 1 mm; no gap; number of slices = 240; TR = 2300 ms; TE = 3 ms; matrix = 256 × 256; FOV = 244 mm; voxel size = 1 × 1 × 1 mm) were acquired with a 3.0 Tesla Siemens Trio MRI system from the Hospital Clinic of Barcelona. The FLAIR images (slice thickness = 5.2 mm; no gap; number of slices = 19; TR = 7295 ms; TE = 12 ms; matrix = 256 × 256; FOV = 230 mm; voxel size = 0.89 × 0.89 × 5.2 mm) were acquired with a 1.5 Philips Intera scan at the University Hospital of Bellvitge. An expert neurologist assessed the MRI images and confirmed that TLE patients had no structural abnormalities except unilateral hippocampal sclerosis. For each participant, the MRI session took around one week before the ERP session.

### Voxel-Based Morphometry (VBM) of hippocampus

To evaluate the differences in Grey Matter volume between patients and controls within the hippocampus, regions of interest (ROIs) for the left and right hippocampi were defined based on the Anatomical Automatic Labelling Atlas in Montreal Neurologic Institute space (MNI) using the WFU pickatlas tool^85–87^. Voxel-Based Morphometry within these ROIs (VBM)^88^ was carried out using Statistical Parametric Mapping software (SPM8; Wellcome Department of Imaging Neuroscience, University College, London, UK, www.fil.ion.ucl.ac.uk/spm). Specifically, New Segment^89^ was applied to the structural T1-weighted images of each subject from both the patient and the control groups. The resulting grey matter (GM) tissue probability maps were imported and fed into Diffeomorphic Anatomical Registration using Exponentiated Lie algebra (DARTEL)^90^ to achieve spatial normalization in MNI space (using “modulation” to compensate for the effect of spatial normalization). Normalized images were smoothed using an isotropic spatial filter (FWHN = 8 mm) to reduce residual inter-individual variability. Individual smoothed GM volume images for controls and left TLE-UHS patients were entered into a two-sample *t*-test and two contrasts of interest were calculated: Controls > LTLE-UHS and LTLE-UHS > Controls. The same analysis was repeated using the controls and the right TLE-UHS patients.

Contrasts were thresholded at a p < 0.005 uncorrected threshold at the voxel level with a cluster extent of more than 50 contiguous voxels^91^. A p < 0.05 Family Wise Error for small volume correction (SVC) was applied to the thresholded images and only clusters with a peak voxel showing significant differences at the corrected threshold are reported.

## Electronic supplementary material

Supplementary information
