# Supplementary material for: Auditory Target and Novelty Processing in Patients with Unilateral Hippocampal Sclerosis: A Current-Source Density Study
Source: Sci Rep. 2017 May 9;7:1612. doi: 10.1038/s41598-017-01531-8 (PMC5431625; doi:10.1038/s41598-017-01531-8)
Supplement: Supplementary file 1 — Supplementary information [file 41598_2017_1531_MOESM1_ESM.pdf]

AUDITORY TARGET AND NOVELTY PROCESSING IN PATIENTS WITH UNILATERAL HIPPOCAMPAL SCLEROSIS: A CURRENT-SOURCE DENSITY STUDY

Adrià Vilà-Balló[1,2,3,*], Clément François[1,2,4,*], David Cucurell[1,2,3], Júlia Miró[1,5], Mercè Falip[5], Montserrat Juncadella[5], Antoni Rodríguez-Fornells[1,2,6]

- Supporting Information –

RESULTS

In order to better delineate the activity related to target processing, we analyzed target-related CSD-transformed Event Related brain Potentials (ERP) and CSD event-related spectral perturbations (ERSP) for the participants with more than 20 correctly responded target trials, and also including only trials with responses. This threshold was selected based on previous report showing that a minimum of 20 correct trials is needed to obtain a reliable ERP component (Marco-Pallares et al., 2011). Accordingly to this criterion, we excluded 4 patients and we analyzed the behavioral and CSD data in 18 healthy controls and 14 temporal-lobe epileptic patients with unilateral sclerotic hippocampus (TLE-UHS). Both groups remained matched for gender (Controls: nine men; TLE-UHS: eight men; $U = 117$, $Z = -.395$, $p = .693$), age, years of education, and handedness (see Tables S3 and S4).

In order to test whether the side of the lesion had a differential effect on target and novelty processing, we performed a complementary analysis including the factor Lesion side as between-subject factor. Specifically, for both behavioral and electrophysiological measures we compared the 9 TLE-UHS patients with a left and the 9 patients with a right hippocampal sclerosis. Both groups remained matched for age,

years of education, and handedness (see Tables S1 and S5). However, a significant difference was found for gender (Left TLE-UHS: two men; Right TLE-UHS: seven men; $U = 18$, $Z = -2.291$, $p = .022$).

Finally, in order to test the effect of GABAergic medication on target and novelty processing, we compared the 8 TLE-UHS patients with medication to the 10 patients without medication. Both groups were matched for gender (medicated group: four women; non-medicated group: five women; $U = 31$, $Z = -.922$, $p = .460$), age, years of education, and handedness (see Tables S1 and S6).

Behavioral results

Controls vs. good responders patients

At the behavioral level, the control group had faster mean RTs for targets (494.2 ± 66.5 ms) than the TLE-UHS group (551.6 ± 85.7 ms; $t(30) = -2.133$, $p = .041$). Importantly, we did not observe any significant differences between groups for the percentage of non-responded trials (controls: 12.0 % ± 13.8; TLE-UHS: 17.1 % ± 17.8; $t(30) = -.908$, $p = .371$). Similar results were obtained for the percentage of responses for non-target trials (controls: 9.3 % ± 12.2; patients: 11.3 % ± 6.1; $t(30) = -.568$, $p = .574$).

Left vs. Right

No differences were observed between the left and right TLE-UHS patients in any of the behavioral measures with similar RTs (left: 555.6 ± 70.9 ms; right: 546.6 ± 104.5 ms; $t(12) = .210$, $p = .837$), similar percentage of non-responded trials (left: 33.9 % ± 38.5; right: 36.4 % ± 40.2; $t(16) = -.138$, $p = .892$) and percentage of responses for non-target trials (left: 12.3 % ± 6.7; right: 13.0 % ± 7.3; $t(16) = -.210$, $p = .836$).

Medicated vs. Non-medicated

The TLE-UHS patients with GABAergic medication were slower than patients without medication (non-medicated patients: 513.2 ± 75.2 ms; medicated patients: 602.6 ± 75.3 ms; $t(12) = -2.200$, $p = .048$). No effects of medication were found for the percentage of non-responded trials (non-medicated patients: 28.5 % ± 12.6; medicated patients: 43.5 % ± 36.8; $t(16) = -.820$, $p = .424$) and percentage of responses for non-target trials (non-medicated patients: 13.8 % ± 7.4; medicated patients: 11.1 % ± 6.3; $t(16) = .830$, $p = .419$).

Time analysis of CSD waveforms

Controls vs. good responders patients

Figure S1 shows the CSD waveforms for target and novelty stimuli.

For the target-related P3b source, results of the ANOVA failed to show significant differences between the two groups (main effect of Group: $F(1,30) = .811$, $p = .375$; Group x electrode interaction: $F(2,60) = .073$, $p = .919$, $\varepsilon = .932$). Similar results were obtained in the peak-to-peak analyses at Pz electrode for the peak amplitude ($t(30) = .089$, $p = .930$), and for the peak latency ($t(30) = -.784$, $p = .439$), therefore indicating that P3b source was similar in the two groups.

For the novelty-related P3a source, results of the ANOVA did not reveal significant differences between groups (main effect of Group: $F(1,30) = 2.371$, $p = .134$; Group x electrode interaction: $F(2,60) = 1.051$, $p = .356$, $\varepsilon = .989$). Similar results were obtained in the peak-to-peak analyses at Fz electrode for the peak amplitude ($t(30) = 1.832$, $p = .078$), and for the peak latency ($t(30) = -1.436$, $p = .161$), indicating that the P3a source was similar in the two groups.

Left vs. Right

Figure S2 shows the CSD waveforms for both groups of patients (left- and right-lesioned). No significant effect of lesion side was found for the target-related P3b source (main effect of lesion side: $F(1,16) = 1.324$, $p = .267$; lesion side x electrode interaction: $F(2,32) = .147$, $p = .826$, $\varepsilon = .832$). Similarly, the peak-to-peak analysis discarded any effect of lesion side for the peak mean amplitude (Left-lesioned group: 32.59 ± 14.22 $\mu V/cm^2$, Right-lesioned group: 28.73 ± 9.00 $\mu V/cm^2$; $t(16) = .689$, $p = .501$) nor for the peak latency (Left-lesioned group: 551.11 ± 64.18 ms, Right-lesioned group: 501.78 ± 52.31 ms; $t(16) = 1.787$, $p = .093$), therefore indicating that the target-related P3b source was not affected by the side of the lesion.

For the novelty-related P3a source, no significant effect of lesion side was found (main effect of lesion side: $F(1,16) = 1.44$, $p = .709$; lesion side x electrode interaction: $F(1,32) = .980$, $p = .365$, $\varepsilon = .729$). As for the P3b source, no significant effect of lesion side was observed for peak mean amplitude (Left-lesioned group: 37.11 ± 8.45 $\mu V/cm^2$, Right-lesioned group: 33.41 ± 17.06 $\mu V/cm^2$; $t(16) = .584$, $p = .568$) nor for the peak latency (Left-lesioned group: 340.89 ± 41.17 ms, Right-lesioned group: 338.67 ± 44.68 ms; $t(16) = .110$, $p = .914$), indicating that the novelty-related P3a source was not affected by the side of the lesion.

Medicated vs. Non-medicated

Figure S3 shows the CSD waveforms for patients with and without medication. For the target-related P3b source, no differences were found in the mean amplitude (main effect of group: $F(1,16) < .001$, $p = .991$; group x electrode interaction: $F(2,32) = .449$, $p = .607$, $\varepsilon = .828$). Similar results were obtained in the peak-to-peak analysis for peak mean amplitude (Control: 33.35 ± 9.42 $\mu V/cm^2$, TLE-UHS: 27.30 ± 14.00

μV/cm^2; $t(16) = 1.094$, $p = .290$). However and in line with the behavioral data, this analysis revealed differences in peak latency with medicated patients exhibiting a delayed P3b peak than non-medicated patients (Non-medicated group: 497.60 ± 55.78 ms, Medicated group: 562.50 ± 52.19 ms; $t(16) = -2.522$, $p = .022$).

Regarding the novelty-related P3a source, no effect of medication was observed in the mean amplitude (main effect of group: $F(1,16) = .004$, $p = .953$; group x electrode interaction: $F(1,32) = .050$, $p = .918$, ε = .787). No significant effect of medication was observed for peak mean amplitude (Non-medicated group: 36.03 ± 17.03 μV/cm^2, Medicated group: 34.29 ± 6.92 μV/cm^2; $t(16) = .272$, $p = .789$) nor for peak latency (Non-medicated group: 345.20 ± 41.36 ms, Medicated group: 333.00 ± 43.91 ms; $t(16) = .605$, $p = .553$).

Time-Frequency analysis of CSD waveforms

Controls vs. good responders patients

Figure S4 shows the ERSP in both groups and for the different types of stimuli.

As done for the entire group of participants, differences in power between target-standard and novel-standard were compared between controls and patients for the theta, low-beta and alpha bands. For target stimuli, patients exhibited significantly lesser theta ERS than controls (main effect of group: $F(1,30) = 17.665$, $p < .001$; group x electrode interaction: $F(2,60) = 12.615$, $p < .001$, ε = .776). This difference was significant at Cz and Pz electrodes (Fz: $t(30) = 1.078$, $p = .229$; Cz: $t(30) = 4.646$, $p < .001$; Pz: $t(30) = 6.085$, $p < .001$). No significant differences were observed for the low-beta ERS (main effect of group: $F(1,30) = 1.307$, $p = .262$; group x electrode interaction: $F(2,60) = .549$, $p = .560$, ε = .889), nor for the alpha ERD (main effect of group: $F(1,30) = 2.469$, $p = .125$; group x electrode interaction: $F(2,60) = 1.127$, $p = .323$, ε = .816).

For novel stimuli, patients exhibited significantly lesser theta ERS than controls (main effect of group: $F(1,30) = 21.657$, $p < .001$; group x electrode interaction $F(2,60) = 5.304$, $p = .012$, $\varepsilon = .817$). This difference was largest over Cz and Pz electrodes (Fz: $t(30) = 3.700$, $p = .001$; Cz: $t(30) = 3.919$, $p < .001$; Pz: $t(30) = 5.226$, $p < .001$). Similarly, patients showed significantly lesser low-beta ERS than controls (main effect of group: $F(1,30) = 7.646$, $p = .010$; group x electrode interaction $F(2,60) = .547$, $p = .553$, $\varepsilon = .844$). Regarding the alpha ERD, no differences between patients and controls were found (main effect of group: $F(1,30) = .176$, $p = .682$; group x electrode interaction: $F(2,60) = .528$, $p = .550$, $\varepsilon = .760$).

Left vs. Right

Figure S5 shows the ERSP plots for both groups of patients (left- and right-lesioned). The differences of power between target stimuli and standards and (ii) between novel stimuli and standards were compared between left and right lesioned patients for the theta, low-beta, and alpha bands.

For target stimuli, no significant effect of lesion side was observed for theta ERS (main effect of lesion side: $F(1,16) = .391$, $p = .541$; and lesion side x electrode interaction; $F(2,32) = 1.186$, $p = .296$, $\varepsilon = .535$) nor for low-beta ERS (main effect of lesion side: $F(1,16) = .992$, $p = .334$; lesion side x electrode interaction: $F(2,32) = 1.542$, $p = .234$, $\varepsilon = .624$). However, left-lesioned patients exhibited greater alpha ERD than right-lesioned patients (main effect of Lesion side: $F(1,16) = 6.180$, $p = .024$; lesion side x electrode interaction: $F(2,32) = .098$, $p = .800$, $\varepsilon = .595$).

Regarding novel stimuli, we did not find a significant effect of lesion side for theta ERS (main effect of lesion side: $F(1,16) = 1.599$, $p = .224$; lesion side x electrode interaction: $F(2,32) = .088$, $p = .863$, $\varepsilon = .747$), nor for low-beta ERS (main effect of

lesion side: $F(1,16) = .422$, $p = .525$; lesion side x electrode interaction: $F(2,32) = 1.049$, $p = .344$, $\varepsilon = .714$), nor for alpha ERD (main effect of lesion side: $F(1,16) = 1.279$, $p = .275$; lesion side x electrode interaction: $F(2,32) = 1.128$, $p = .310$, $\varepsilon = .553$).

Medicated vs. Non-medicated

Figure S6 shows the ERSP plots for patients with and without GABAergic medication. The differences of power between (i) target stimuli and standards and (ii) between novel stimuli and standards were compared between medicated and non-medicated patients for the theta, low-beta, and alpha bands.

For target stimuli, no significant effect of group was found for theta ERS (main effect of group: $F(1,16) = .048$, $p = .829$; group x electrode interaction; $F(2,32) = 1.460$, $p = .246$, $\varepsilon = .536$) nor for low-beta ERS (main effect of group: $F(1,16) = .017$, $p = .897$; group x electrode interaction: $F(2,32) = .697$, $p = .443$, $\varepsilon = .616$), and for alpha ERD (main effect of group: $F(1,16) = .252$, $p = .623$; group x electrode interaction: $F(2,32) = 1.163$, $p = .305$, $\varepsilon = .583$).

For novel stimuli, no significant effect of group was observed for theta ERS (main effect of group: $F(1,16) = .182$, $p = .675$; group x electrode interaction: $F(2,32) = .862$, $p = .406$, $\varepsilon = .749$), for low-beta ERS (main effect of group: $F(1,16) = 1.449$, $p = .246$; group x electrode interaction: $F(2,32) = .566$, $p = .515$, $\varepsilon = .698$), nor for alpha ERD (main effect of group: $F(1,16) = 1.079$, $p = .314$; group x electrode interaction: $F(2,32) = .125$, $p = .760$, $\varepsilon = .567$).

METHOD

Participants

Demographic details for participants (see Table S1) including age, years of education (Educ.), Edinburg Handedness scores (handedness) for TLE and control participants are reported. Additional data is provided regarding TLE participants, such as age at epilepsy onset (Onset), seizure type (Type): complex partial seizures (cps) and the number of drugs prescribed.

Neuropsychological assessment

Neuropsychological data were obtained from both controls and patients. All participants completed a reduced version of the Edinburgh Handedness Inventory[1]; Logical memory I (immediate verbal memory) and II (delayed verbal memory), the Visual reproduction I (immediate visual memory) and II (delayed visual memory), the Digits Span, and the Letters and numbers subtests of the Wechsler Memory Scale III[2]; the Vocabulary (IQ estimation) subtest of the Wechsler Adult Intelligence Scale[3]; the Rey Auditory Verbal Learning Test[4,5], the Trial Making Test (TMT-A and TMT-B[6,7]), the Semantic Fluency and the Phonemic Fluency subtests of the Barcelona Test-R[8]; the Rey-Osterrieth Complex Figure (copy and memory) (RCF)[4,8,9]. For each neuropsychological subtest we used independent two-sample *t*-tests to compute the differences between groups of participants (Controls, TLE-UHS). The mean scores of the neuropsychological data are summarized in Table S2.

REFERENCES

1. Oldfield, R. C. The assessment and analysis of handedness: The Edinburgh inventory. *Neuropsychologia* **9,** 97–113 (1971).
2. Wechsler, D. *WMS-III. Escala de memoria de Wechsler-III.* (TEA, 2004).
3. Wechsler, D. *WAIS III Escala de Inteligencia de Wechsler para Adultos – III.* (TEA, 1999).
4. Rey, A. L'examen psychologique dans les cas d'encéphalopathie traumatique. (Les problems.). [The psychological examination in cases of traumatic encepholopathy. Problems.]. *Arch. Psychol.* **28,** 215–285 (1941).
5. Schmidt, M. *Rey Auditory Verbal Learning Test: RAVLT : a Handbook.* (Western Psychological Services, 1996).
6. Reitan, R. M. The relation of the Trail Making Test to organic brain damage. *J. Consult. Psychol.* **19,** 393–394 (1955).
7. Davies, D. M. The influence of age on trail making test performance. *J. Clin. Psychol.* **24,** 96–98 (1968).
8. Peña-Casanova, J. *Integrated Neuropsychological Exploration Program-Barcelona Test Revised.* (Masson, 2005).
9. Osterrieth, P. A. Le test de copie d'une figure complexe; contribution à l'étude de la perception et de la mémoire. [Test of copying a complex figure; contribution to the study of perception and memory.]. *Arch. Psychol.* **30,** 206–356 (1944).

- Supporting Figures –

Figure S1. Grand mean CSD waveforms for standard (grey line), target (black line) and novel (red line), at midline electrodes (Fz, Cz, and Pz), from −100 to 800 ms, for both the control and good responders patients. The difference waveforms associated to the target minus standard (black line) and novel minus standard (red line) are showed. Grey areas indicate the time-windows considered for the analyses. At the bottom, the voltage distributions of the P3b source (Target minus standard, −24/24μV/cm^2), and of the P3a source (Novel minus standard, −24/24μV/cm^2) are depicted.

Figure S2. Grand mean CSD waveforms for standard (grey line), target (black line) and novel (red line), at midline electrodes (Fz, Cz, and Pz), from −100 to 800 ms, for both the left- and right-lesioned patients. The difference waveforms associated to the target minus standard (black line) and novel minus standard (red line) are showed. Grey areas indicate the time-windows considered for the analyses. At the bottom part of the figure, the voltage distributions of the P3b source (Target minus standard, −24/24μV/cm^2), and of the P3a source (Novel minus standard, −24/24μV/cm^2) are depicted.

Figure S3. Grand mean CSD waveforms for standard (grey line), target (black line) and novel (red line), at midline electrodes (Fz, Cz, and Pz), from −100 to 800 ms, for non-medicated (left) and medicated patients (right). The difference waveforms associated to the target minus standard (black line) and novel minus standard (red line) are showed. Grey areas indicate the time-windows considered for the analyses. At the bottom part of the figure, the scalp distributions of the P3b source (Target minus standard, −24/24μV/cm^2), and of the P3a source (Novel minus standard, −24/24μV/cm^2) are depicted.

Figure S4. Grand mean CSD event-related spectral perturbation representing changes in power with respect to baseline for standard, target, and novel stimuli at midline electrodes (Fz, Cz, and Pz), for the control (left) and good responders patients (right). The increase/decrease of power is represented from −100 to 800 ms. The black squares indicate the time-windows in the different frequency bands considered for the analyses. The differences in power between target and standard, and between novel and standard are depicted at the bottom. The power distributions of theta (4−8 Hz), alpha (8−12 Hz), and low-beta (12−15 Hz) activities for target minus standard and for novel minus standard are depicted.

Figure S5. Grand mean CSD event-related spectral perturbation representing the changes in power with respect to baseline for standard, target, and novel stimuli at midline electrodes, for the left-lesioned (left) and right-lesioned patients (right). The increase/decrease of power is represented from −100 to 800 ms. The black squares indicate the time-windows in the different frequency bands considered for the analyses. The differences in power between target and standard, and between novel and standard are depicted at the bottom. The power distributions of theta (4−8Hz), alpha (8−12 Hz) and low-beta (12−15 Hz) activities for both differences (target minus standard and novel minus standard) are depicted.

Figure S6. Grand mean CSD event-related spectral perturbation representing the changes in power with respect to baseline for standard, target, and novel stimuli at midline electrodes, for non-medicated (left) and medicated patients (right). The increase/decrease of power is represented from −100 to 800 ms. The black squares

indicate the time-windows in the different frequency bands considered for the analyses. The differences in power between target and standard, and between novel and standard are depicted at the bottom. The power distributions of theta (4−8 Hz), alpha (8−12 Hz) and low-beta (12−15 Hz) activities for both differences (target minus standard and novel minus standard) are depicted.

Table S1. Demographic data for TLE patients and controls (left and right) included in the study. Age, years of education (Educ.), Edinburg Handedness scores (Hand), age at epilepsy onset (Onset), seizure type (Type): complex partial seizures (cps). Prescribed number of epileptic drugs (Num. AEDS), benzodiazepine and barbiturates (BZD & BARB).

Code	Group	Age	Gender	Educ.	Hand	Onset	Type	Freq	Num. AEDS	BZD & BARB
T01	TLE-R	45	M	16	18	10M	cpc	1-2/month	4	No
T02	TLE-L	37	F	8	10	14M	cpc	1-2/month	3	clobazam 10mg/d
T03	TLE-R	51	M	11	10	11Y	cpc	1/month	3	clobazam 20mg/d
T04	TLE-R	37	M	11	10	18Y	cpc	4-6/month	3	phenobarbital 100mg/d
T05	TLE-R	50	F	8	10	18Y	cpc	6-8/month	3	phenobarbital 100mg/d
T06	TLE-R	65	M	8	10	41Y	cpc	4/month	2	No
T07	TLE-L	33	F	11	10	16Y	cpc	30-35/month	3	clobazam 10mg/d
T08	TLE-L	32	M	16	10	23Y	cpc	8-10/month	2	No
T09	TLE-R	38	M	16	10	4Y	cpc	2-4/month	2	No
T10	TLE-R	22	M	16	20	17Y	cpc	5/month	2	No
T11	TLE-L	47	F	12	10	13M	cpc	7-9/month	2	No
T12	TLE-L	39	F	14	10	12M	cpc	5-6/month	4	phenobarbital 150mg/d
T13	TLE-L	29	M	14	10	15Y	cpc	3-4/month	3	phenobarbital 200mg/d
T14	TLE-L	25	M	9	10	13Y	cpc	1/month	2	No
T15	TLE-L	50	F	14	10	32Y	cpc	9-10/month	3	clobazam 25mg/d
T16	TLE-R	33	F	12	10	21Y	cpc	2/month	2	No
T17	TLE-L	46	F	14	10	8Y	cpc	18-20/month	2	No
T18	TLE-L	36	F	17	10	2Y	cpc	4-6/month	2	No
C01	Control	49	M	15	13					
C02	Control	42	F	10	10					
C03	Control	48	M	11	10					
C04	Control	39	M	10	15					
C05	Control	35	F	16	10					
C06	Control	53	F	8	10					
C07	Control	61	M	10	38					
C08	Control	25	F	17	10					
C09	Control	30	M	8	10					
C10	Control	35	M	10	10					
C11	Control	25	M	17	10					
C12	Control	43	F	10	10					
C13	Control	43	F	10	10					
C14	Control	29	F	18	10					
C15	Control	28	M	12	10					
C16	Control	21	M	13	10					
C17	Control	51	F	12	10					
C18	Control	48	F	16	10					

Table S2. Demographic data for controls and patients, included in the study. Age, years of education (Educ). Mean scores of neuropsychological data for controls and patients, included in the study. The neuropsychological measures are Edinburgh Handedness Inventory (Handedness), LMI (Logical Memory I), LMII (Logical Memory II), VRI (Visual Reproduction I), VRII (Visual Reproduction II), Dig_span (Digits Span), Letter num. (Letters and numbers), RV. A5 (RAVLT total learning at trial 5), RV. A6 (RAVLT immediate recall), RV. A7 (RAVLT delayed recall), RV. Rcog. (RAVLT recognition), TMT A (Trial Making Test A), TMT B (Trial Making Test B), Voc (Vocabulary), BNT (Boston Naming Test), Flue.(s) (Semantic Fluency), and Flue.(p) (Phonemic Fluency); RCF (Rey-Osterrieth Complex Figure copy), RCF Time (RCF copy time), and RCF recall (RCF immediate recall). The *t*-tests comparing the control group *Vs*. the TLE-UHS are given. Significant FDR-corrected differences are indicated.

	**Controls**		**TLE-UHS**			
	**M**	**SD**	**M**	**SD**	***t***	***P***
**Age**	39.2	11.3	39.7	10.6	-0.2	0.880
**Educ.**	12.3	3.2	12.6	3.1	-0.3	0.792
**Handedness**	12.0	6.6	11.0	2.9	0.6	0.562
**LMI**	36.7	12.7	30.4	9.5	1.7	0.100
**VRI**	93.3	12.2	79.7	18.6	2.6	0.014*
**LMII**	23.1	9.4	16.1	7.4	2.5	0.018*
**VRII**	82.2	15.3	57.3	24.1	3.7	0.001*
**Dig. span**	16.3	4.4	14.1	6.2	1.2	0.234
**Letter num.**	10.3	3.1	9.1	2.3	1.2	0.226
**RV. A5**	13.7	1.1	12.2	2.2	2.6	0.016*
**RV. A6**	12.8	2.1	9.5	3.2	3.6	0.001*
**RV. A7**	12.8	1.9	9.4	3.3	3.8	0.001*
**RV. Rcog.**	14.5	0.6	13.1	2.1	2.7	0.012*
**TMT A**	42.3	21.7	44.9	18.8	-0.4	0.702
**TMT B**	93.1	58.9	106.8	63.5	-0.7	0.515
**Voc.**	47.1	7.8	37.5	6.9	3.9	<0.001*
**BNT**	54.8	3.6	49.8	6.2	3.0	0.006*
**Flue. (s)**	21.7	4.7	18.7	5.7	1.7	0.094
**Flue. (p)**	17.2	4.8	14.9	7.6	1.1	0.301
**RCF**	34.8	2.0	31.4	7.0	1.9	0.071
**RCF Time**	134.1	49.0	172.2	66.3	-1.9	0.061
**RCF Recall**	24.2	5.1	16.2	7.2	3.8	0.001*

*$p < 0.05$ FDR-corrected

Table S3. Demographic data for the good performers TLE patients and controls included in the supplemental analyses. Age, years of education (Educ.), Edinburg Handedness scores (Hand), age at epilepsy onset (Onset), seizure type (Type): complex partial seizures (cps). Prescribed number of epileptic drugs (Num. AEDS), benzodiazepine and barbiturates (BZD & BARB).

Code	Group	Age	Gender	Educ.	Hand	Onset	Type	Freq	Num. AEDS	BZD & BARB
T01	TLE-R	45	M	16	18	10M	cpc	1-2/month	4	No
T02	TLE-L	37	F	8	10	14M	cpc	1-2/month	3	clobazam 10mg/d
T03	TLE-R	51	M	11	10	11Y	cpc	1/month	3	clobazam 20mg/d
T04	TLE-R	37	M	11	10	18Y	cpc	4-6/month	3	phenobarbital 100mg/d
T08	TLE-L	32	M	16	10	23Y	cpc	8-10/month	2	No
T09	TLE-R	38	M	16	10	4Y	cpc	2-4/month	2	No
T10	TLE-R	22	M	16	20	17Y	cpc	5/month	2	No
T11	TLE-L	47	F	12	10	13M	cpc	7-9/month	2	No
T12	TLE-L	39	F	14	10	12M	cpc	5-6/month	4	phenobarbital 150mg/d
T13	TLE-L	29	M	14	10	15Y	cpc	3-4/month	3	phenobarbital 200mg/d
T14	TLE-L	25	M	9	10	13Y	cpc	1/month	2	No
T15	TLE-L	50	F	14	10	32Y	cpc	9-10/month	3	clobazam 25mg/d
T16	TLE-R	33	F	12	10	21Y	cpc	2/month	2	No
T18	TLE-L	36	F	17	10	2Y	cpc	4-6/month	2	No
C01	Control	49	M	15	13					
C02	Control	42	F	10	10					
C03	Control	48	M	11	10					
C04	Control	39	M	10	15					
C05	Control	35	F	16	10					
C06	Control	53	F	8	10					
C07	Control	61	M	10	38					
C08	Control	25	F	17	10					
C09	Control	30	M	8	10					
C10	Control	35	M	10	10					
C11	Control	25	M	17	10					
C12	Control	43	F	10	10					
C13	Control	43	F	10	10					
C14	Control	29	F	18	10					
C15	Control	28	M	12	10					
C16	Control	21	M	13	10					
C17	Control	51	F	12	10					
C18	Control	48	F	16	10					

Table S4. Demographic data for the good performers patients and controls included in the supplemental analyses. Age, years of education (Educ). Mean scores of neuropsychological data for controls and patients. The neuropsychological measures are Edinburgh Handedness Inventory (Handedness), LMI (Logical Memory I), LMII (Logical Memory II), VRI (Visual Reproduction I), VRII (Visual Reproduction II), Dig_span (Digits Span), Letter num. (Letters and numbers), RV. A5 (RAVLT total learning at trial 5), RV. A6 (RAVLT immediate recall), RV. A7 (RAVLT delayed recall), RV. Rcog. (RAVLT recognition), TMT A (Trial Making Test A), TMT B (Trial Making Test B), Voc (Vocabulary), BNT (Boston Naming Test), Flue.(s) (Semantic Fluency), and Flue.(p) (Phonemic Fluency); RCF (Rey-Osterrieth Complex Figure copy), RCF Time (RCF copy time), and RCF recall (RCF immediate recall). The *t*-tests comparing the control group *Vs*. the TLE-UHS are given. Significant FDR-corrected differences are indicated.

	**Controls**		**TLE-UHS**			
	**M**	**SD**	**M**	**SD**	***t***	***P***
**Age**	39.2	11.3	37.2	8.8	.5	.599
**Educ.**	12.3	3.2	13.3	2.8	-.9	.389
**Handedness**	12.0	6.6	11.3	3.3	.4	.715
**LMI**	36.7	12.7	32.4	9.7	1.1	.295
**VRI**	93.3	12.2	86.4	12.7	1.6	.126
**LMII**	23.1	9.4	17.9	7.3	1.7	.095
**VRII**	82.2	15.3	64.4	18.0	3.0	.005*
**Dig. span**	16.3	4.3	15.0	6.8	.6	.521
**Letter num.**	10.3	3.1	9.6	2.3	.7	.486
**RV. A5**	13.7	1.1	12.8	1.8	1.7	.102
**RV. A6**	12.8	2.1	10.5	2.9	2.6	.015
**RV. A7**	12.8	1.9	10.5	2.8	2.8	.009
**RV. Rcog.**	14.5	.6	13.7	1.4	1.9	.073
**TMT A**	42.3	21.7	37.7	13.2	.7	.489
**TMT B**	93.1	58.9	84.0	31.9	.5	.606
**Voc.**	47.1	7.8	38.6	6.9	3.2	.003*
**BNT**	54.8	3.6	51.4	5.1	2.2	.033
**Flue. (s)**	21.7	4.7	19.9	5.7	1.0	.317
**Flue. (p)**	17.2	4.8	15.5	8.3	.7	.478
**RCF**	34.8	2.0	33.4	3.1	1.5	.161
**RCF Time**	134.1	49.0	156.8	54.0	-1.2	.231
**RCF Recall**	24.2	5.1	18.2	6.9	2.8	.009*

*p < 0.05 FDR-corrected

Table S5. Demographic data for left and right patients included in the supplemental analyses. Age, years of education (Educ). Mean scores of neuropsychological data for left and right lesion patients. The neuropsychological measures are Edinburgh Handedness Inventory (Handedness), LMI (Logical Memory I), LMII (Logical Memory II), VRI (Visual Reproduction I), VRII (Visual Reproduction II), Dig_span (Digits Span), Letter num. (Letters and numbers), RV. A5 (RAVLT total learning at trial 5), RV. A6 (RAVLT immediate recall), RV. A7 (RAVLT delayed recall), RV. Rcog. (RAVLT recognition), TMT A (Trial Making Test A), TMT B (Trial Making Test B), Voc (Vocabulary), BNT (Boston Naming Test), Flue.(s) (Semantic Fluency), and Flue.(p) (Phonemic Fluency); RCF (Rey-Osterrieth Complex Figure copy), RCF Time (RCF copy time), and RCF recall (RCF immediate recall). The *t*-tests comparing the left and right are given. No significant differences have been found after the FDR-correction.

	Left TLE-UHS		Right TLE-UHS			
	**M**	**SD**	**M**	**SD**	***t***	***P***
**Age**	38.0	8.4	41.4	12.8	-.7	.509
**Educ.**	12.6	2.8	12.7	3.4	-.1	.941
**Handedness**	10.0	.0	12.0	4.0	-1.5	.172
**LMI**	30.2	11.2	30.6	8.1	-.1	.943
**VRI**	81.9	15.5	77.4	22.0	.5	.627
**LMII**	15.3	8.3	16.8	6.9	-.4	.693
**VRII**	61.1	16.4	53.6	30.5	.7	.525
**Dig. span**	13.2	4.1	15.0	8.0	-.6	.560
**Letter num.**	8.2	2.2	10.1	2.2	-1.8	.095
**RV. A5**	12.2	2.0	12.1	2.5	.1	.918
**RV. A6**	9.3	3.0	9.7	3.6	-.2	.833
**RV. A7**	8.7	2.6	10.1	4.0	-.9	.376
**RV. Rcog.**	13.7	1.4	12.6	2.5	1.2	.264
**TMT A**	46.2	16.6	43.7	21.6	.3	.782
**TMT B**	113.9	68.9	98.8	60.5	.5	.639
**Voc.**	37.3	6.7	37.7	7.4	-.1	.922
**BNT**	47.4	6.6	52.1	5.2	-1.7	.114
**Flue. (s)**	16.6	4.6	20.9	6.1	-1.7	.110
**Flue. (p)**	16.7	9.6	13.2	5.0	1.0	.352
**RCF**	32.0	4.5	30.8	9.3	.3	.738
**RCF Time**	174.0	68.7	170.1	68.2	.1	.909
**RCF Recall**	19.9	6.9	12.1	5.2	2.6	.019

*$p < 0.05$ FDR-corrected

Table S6. Demographic data for patients with and without GABAergic medication included in the supplemental analyses. Age, years of education (Educ). Mean scores of neuropsychological data for patients with and without GABAergic medication. The neuropsychological measures are Edinburgh Handedness Inventory (Handedness), LMI (Logical Memory I), LMII (Logical Memory II), VRI (Visual Reproduction I), VRII (Visual Reproduction II), Dig_span (Digits Span), Letter num. (Letters and numbers), RV. A5 (RAVLT total learning at trial 5), RV. A6 (RAVLT immediate recall), RV. A7 (RAVLT delayed recall), RV. Rcog. (RAVLT recognition), TMT A (Trial Making Test A), TMT B (Trial Making Test B), Voc (Vocabulary), BNT (Boston Naming Test), Flue.(s) (Semantic Fluency), and Flue.(p) (Phonemic Fluency); RCF (Rey-Osterrieth Complex Figure copy), RCF Time (RCF copy time), and RCF recall (RCF immediate recall). The *t*-tests comparing both groups are given. No significant differences have been found after the FDR-correction.

	**Non-taking GABAergic**		**Taking GABAergic**			
	**M**	**SD**	**M**	**SD**	***t***	***P***
**Age**	40.8	8.5	38.9	12.5	.4	.726
**Educ.**	11.4	2.5	13.6	3.2	-1.6	.128
**Handedness**	10.0	.0	11.8	3.8	-1.5	.171
**LMI**	30.4	5.2	30.4	12.2	.0	.996
**VRI**	80.3	15.1	79.2	21.8	.1	.909
**LMII**	14.9	3.8	17.0	9.5	-.6	.531
**VRII**	47.5	21.3	65.2	24.2	-1.6	.124
**Dig. span**	12.6	4.6	15.3	7.3	-.9	.381
**Letter num.**	8.3	2.2	9.9	2.3	-1.5	.156
**RV. A5**	12.5	1.3	11.9	2.7	.6	.550
**RV. A6**	9.5	2.1	9.5	4.0	.0	1.000
**RV. A7**	9.1	3.0	9.6	3.7	-.3	.775
**RV. Rcog.**	13.1	1.6	13.1	2.4	.0	.980
**TMT A**	52.3	16.4	39.1	19.2	1.5	.144
**TMT B**	136.6	82.4	80.2	21.1	1.9	.097
**Voc.**	35.9	4.4	38.8	8.4	-.9	.385
**BNT**	45.8	7.1	53.0	2.9	-2.7	.024
**Flue. (s)**	15.9	4.2	21.0	5.9	-2.1	.056
**Flue. (p)**	15.3	8.5	14.7	7.3	.1	.884
**RCF**	33.4	2.7	29.7	9.2	1.1	.296
**RCF Time**	176.6	75.4	168.2	61.5	.3	.804
**RCF Recall**	16.3	6.1	16.2	8.5	.0	.968

*$p < 0.05$ FDR-corrected

–Figure S1–

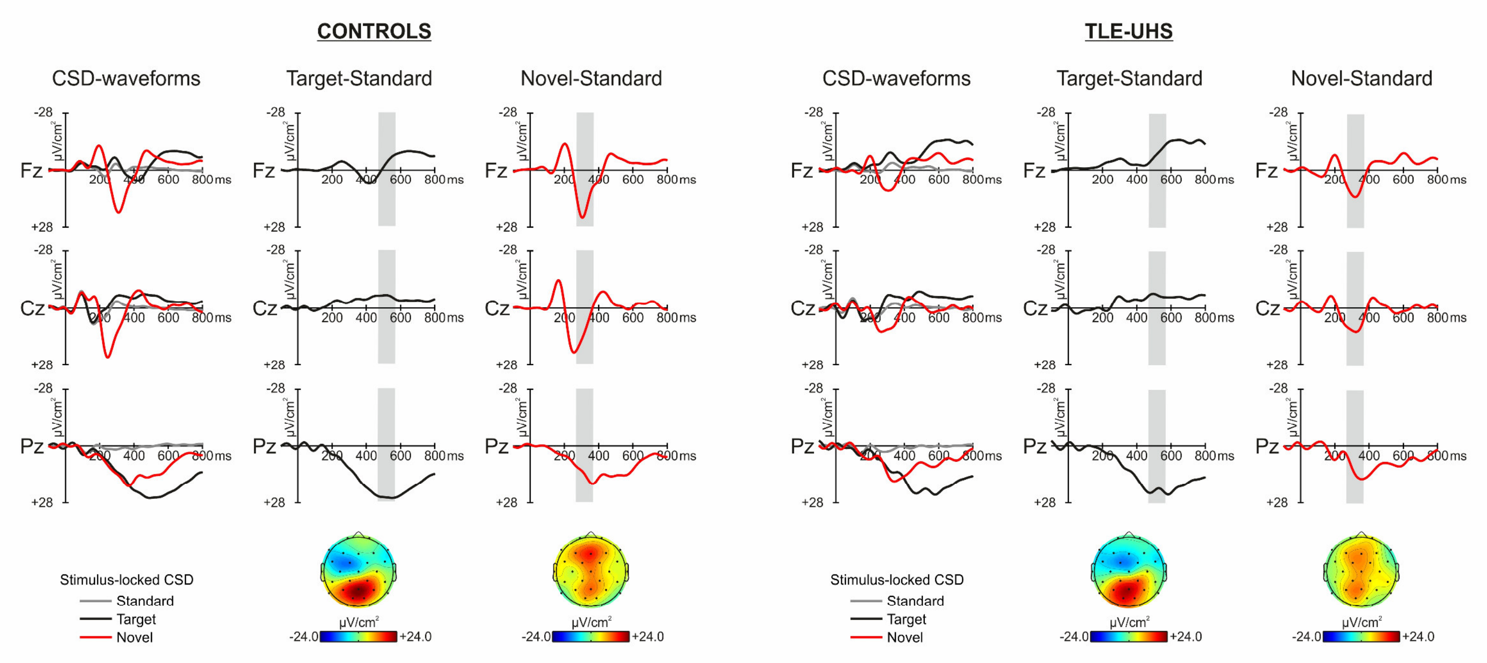
CONTROLS
TLE-UHS
CSD-waveforms
Target-Standard
Novel-Standard
Fz
Cz
Pz
-28
+28
µV/cm²
200
400
600
800ms
Stimulus-locked CSD
Standard
Target
Novel
µV/cm²
-24.0
+24.0

–Figure S2–

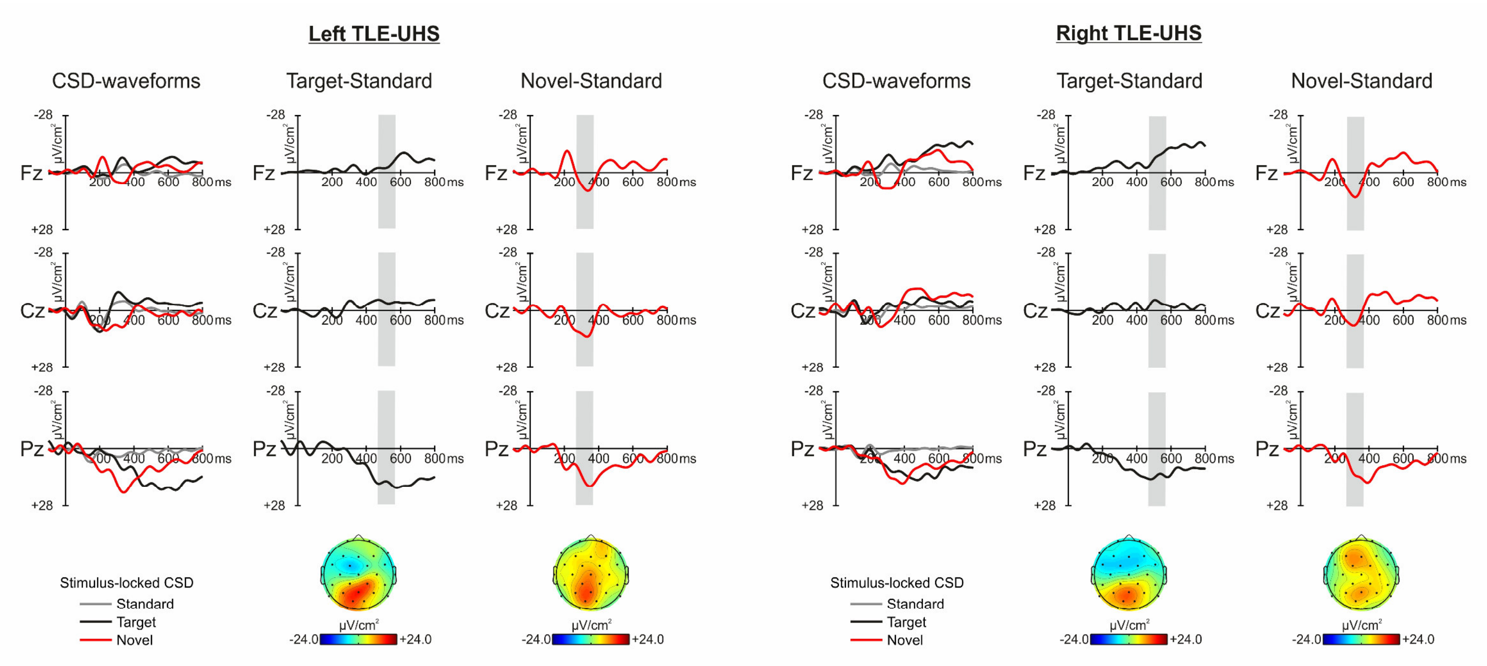
Left TLE-UHS
Right TLE-UHS
CSD-waveforms
Target-Standard
Novel-Standard
Fz
Cz
Pz
-28
+28
μV/cm²
200 400 600 800ms
Stimulus-locked CSD
Standard
Target
Novel
-24.0
+24.0

–Figure S3–

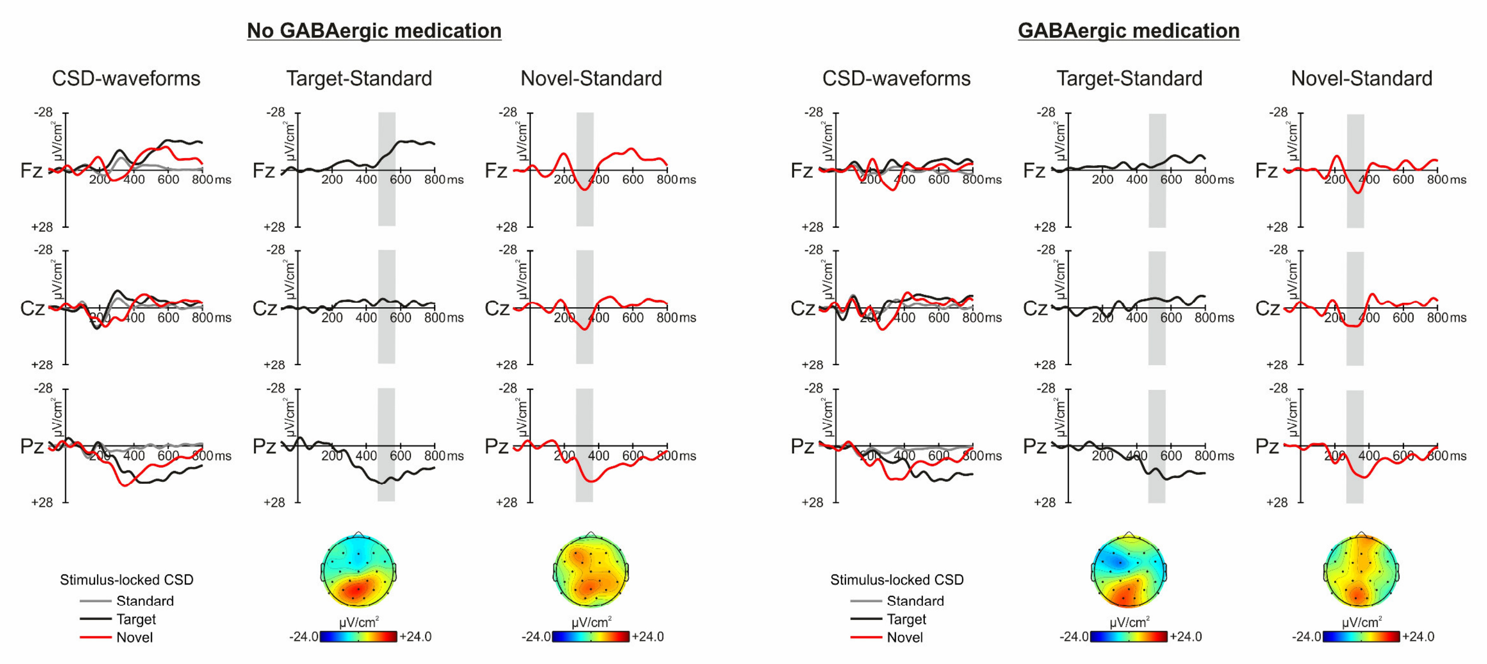
No GABAergic medication
GABAergic medication
CSD-waveforms
Target-Standard
Novel-Standard
Fz
Cz
Pz
-28
+28
μV/cm²
200 400 600 800ms
Stimulus-locked CSD
Standard
Target
Novel
-24.0
+24.0

–Figure S4–

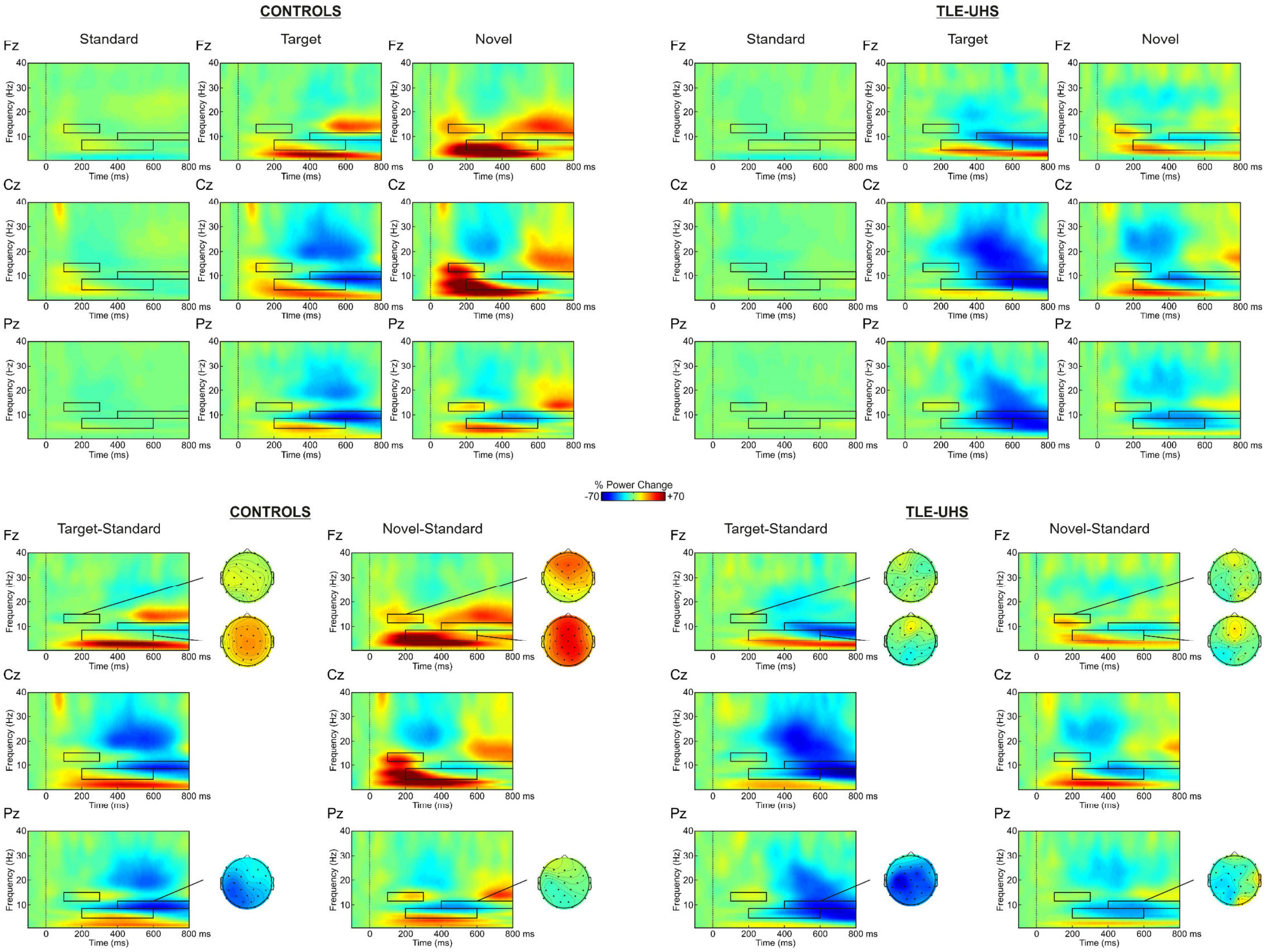

CONTROLS
TLE-UHS
Standard
Target
Novel
Fz
Cz
Pz
Frequency (Hz)
Time (ms)
40
30
20
10
0
200
400
600
800 ms
% Power Change
-70
+70
CONTROLS
TLE-UHS
Target-Standard
Novel-Standard

–Figure S5–

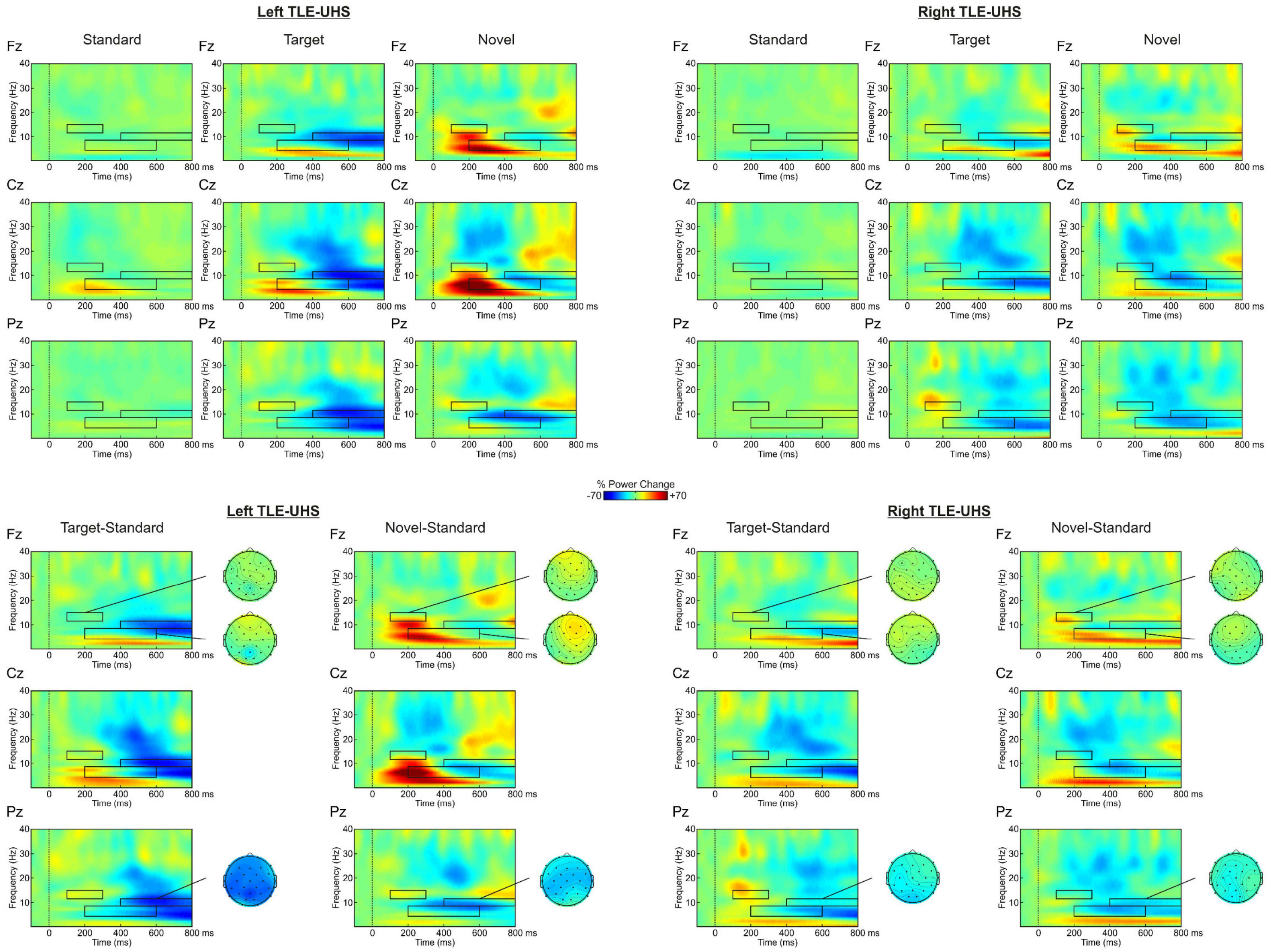
Left TLE-UHS
Right TLE-UHS
Standard
Target
Novel
Fz
Cz
Pz
Frequency (Hz)
Time (ms)
0
200
400
600
800 ms
10
20
30
40
% Power Change
-70
+70
Target-Standard
Novel-Standard

–Figure S6–

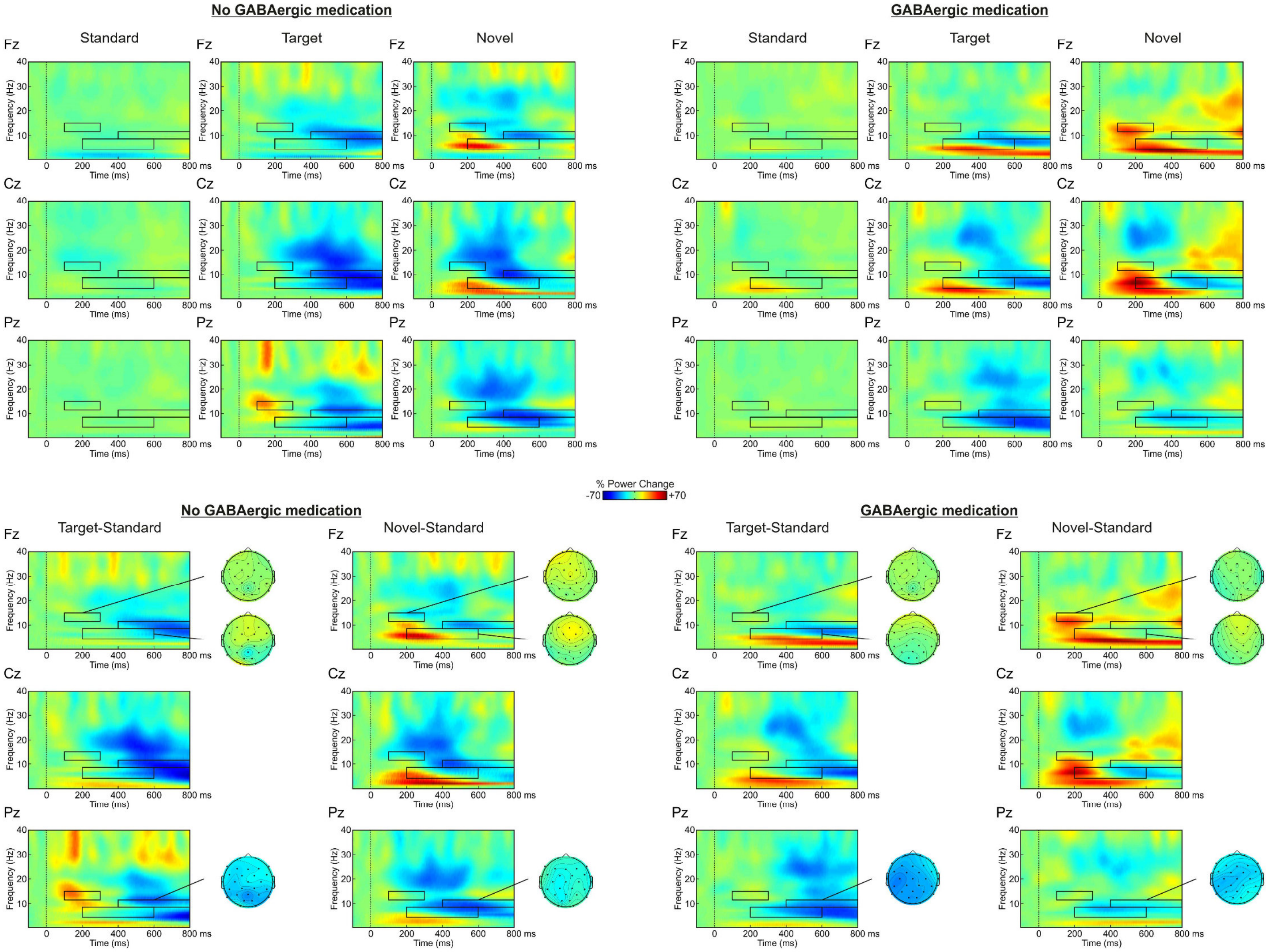

No GABAergic medication
GABAergic medication
Standard
Target
Novel
Fz
Cz
Pz
Frequency (Hz)
Time (ms)
% Power Change
-70
+70
Target-Standard
Novel-Standard
